# Systemic vasculopathy with altered vasoreactivity in a transgenic mouse model of scleroderma

**DOI:** 10.1186/ar2986

**Published:** 2010-04-15

**Authors:** Emma C Derrett-Smith, Audrey Dooley, Korsa Khan, Xu Shi-wen, David Abraham, Christopher P Denton

**Affiliations:** 1 Centre for Rheumatology and Connective Tissue Diseases, UCL Medical School, Royal Free Campus, Rowland Hill Street, London, NW3 2PF, UK

## Abstract

**Introduction:**

Vasculopathy, including altered vasoreactivity and abnormal large vessel biomechanics, is a hallmark of systemic sclerosis (SSc). However, the pathogenic link with other aspects of the disease is less clear. To assess the potential role of transforming growth factor beta (TGF-β) overactivity in driving these cardiovascular abnormalities, we studied a novel transgenic mouse model characterized by ligand-dependent activation of TGF-β signaling in fibroblasts.

**Methods:**

The transgenic mouse strain Tβ RIIΔk-fib is characterized by balanced ligand-dependent upregulation of TGF-β signaling. Aortic and cardiac tissues were examined with histologic, biochemical, and isolated organ bath studies. Vascular and perivascular architecture was examined by hematoxylin and eosin (H&E) and special stains including immunostaining for TGF-β1 and phospho-Smad2/3 (pSmad2/3). Confirmatory aortic smooth muscle cell proliferation, phenotype, and functional assays, including signaling responses to exogenous TGF-β and endothelin-1, were performed. Aortic ring contractile responses to direct and receptor-mediated stimulation were assessed.

**Results:**

Aortic ring contractility and relaxation were diminished compared with wild-type controls, and this was associated with aortic adventitial fibrosis confirmed histologically and with Sircol assay. TGF-β1 and pSmad 2/3 expression was increased in the adventitia and smooth muscle layer of the aorta. Aortic smooth muscle cells from transgenic animals showed significant upregulation of TGF-β- responsive genes important for cytoskeletal function, such as transgelin and smoothelin, which were then resistant to further stimulation with exogenous TGF-β1. These cells promoted significantly more contraction of free floating type I collagen lattices when compared with the wild-type, but were again resistant to exogenous TGF-β1 stimulation. Aortic ring responses to receptor-mediated contraction were reduced in the transgenic animals. Specifically, bosentan reduced endothelin-mediated contraction in wild-type animals, but had no effect in transgenic animals, and endothelin axis gene expression was altered in transgenic animals. Transgenic mice developed cardiac fibrosis.

**Conclusions:**

The histologic, biochemical, and functional phenotype of this transgenic mouse model of scleroderma offers insight into the altered biomechanical properties previously reported for large elastic arteries in human SSc and suggests a role for perturbed TGF-β and endothelin activity in this process.

## Introduction

Vasculopathy is a key pathologic feature of systemic sclerosis (SSc) and leads to important clinical complications including pulmonary arterial hypertension (PAH), scleroderma renal crisis (SRC), and severe Raynaud phenomenon with digital ischemia and infarction. In this study, we explored systemic vasculopathy and cardiovascular abnormalities in a transforming growth factor-beta (TGF-β)-dependent transgenic mouse model that has been previously shown to replicate the skin and lung fibrosis of SSc.

Although many previous studies highlighted microvascular abnormalities in SSc [1], a growing body of evidence exists for structural and functional abnormalities in the macrovascular circulation. Altered large vessel vasoreactivity and abnormal biomechanical properties have been described, including vessel stiffness and elasticity of the aorta and carotid arteries, and impaired flow-mediated dilatation in brachial arteries [2-6]. Although arterial stiffness is usually considered to result in hypertension and an increased propensity to atherosclerosis and aortic aneurysm, none of these is a prevalent feature in SSc [7]. By analogy, TGF-β overactivity is implicated in the pathogenesis of hypertensive arteriosclerosis, SSc, and some inherited vascular diseases that affect aortic structure and function, including Marfan syndrome and Loeys-Dietz syndrome [8-11].

We previously described a novel genetically determined transgenic mouse strain in which ligand-dependent activation of TGF-β signaling occurs selectively in fibroblasts (Tβ RIIΔk-fib). Expression of this kinase-deficient type II TGF-β receptor at low levels facilitates activation of the endogenous type I TGF-β receptor, at least in part by increasing levels of wild-type Tβ RII. Downstream consequences include upregulation of TGF-β1 and other gene products that promote TGF-β activity or activate the latent TGF-β complex. This results in net activation of TGF-β signaling. However, in response to TGF-β1, significant elevation of transgene expression is found. Higher level transgene expression is inhibitory and blocks signaling. Thus, for transcripts upregulated at early time points by TGF-β1, a transient response occurs in transgenic cells, but for transcripts induced at 12 hours when the transgene is also upregulated, suppression is observed. High level transgene expression does not suppress the fibroblast-specific promoter completely, suggesting that other TGF-β-independent pathways also govern the activity of this lineage-specific construct. We have described this as a model of balanced TGF-β upregulation occurring selectively in fibroblasts [12-15].

In the present study, we explored the potential link between TGF-β overactivity and systemic cardiovascular features of SSc. Our results show upregulation of TGF-β signalling pathways and vessel wall fibrosis in the systemic arterial circulation, altered vasoreactivity, and a TGF-β- activated smooth muscle cell phenotype with additional perturbation of endothelin axis signaling. Our work provides support for altered TGF-β activity playing a pivotal role in vasculopathy in this strain and in SSc.

## Materials and methods

### Generation of transgenic mice

The generation and characterization of Tβ RIIΔk-fib transgenic mice were described previously [15]. All animal procedures were conducted in compliance with institutional and national guidelines and with ethics committee approval. Neonatal pups were genotyped by PCR analysis of genomic DNA extracted from tail-biopsy specimens, by using primers specific for the β-galactosidase reporter gene (5'-CGGATAAACGGAACTGGAAA-3' and 5'-TAATCACGACTCGCTGTATC-3') (Sigma-Genosys, Haverhill, UK).

### Histologic analysis

Thoracic aortic and cardiac tissue from sacrificed adult transgenic and littermate sex-matched wild-type mice were dissected and immersed in 10% normal saline or were snap-frozen in liquid nitrogen. Formalin-fixed, paraffin-embedded composite 3 μm sections were mounted onto poly-L-lysine-coated slides (VWR, UK) and stained with H&E, picrosirius red, Elastin van Gieson (EVG), Masson trichrome, and for immunohistochemistry according to standard protocols [16]. Primary antibodies were as follows: CD34 (Abcam, Cambridge, UK); TGF-β1 and pSmad2/3 (Santa Cruz Biotechnology, Santa Cruz, CA); LAP(TGFβ1) (R&D Biosystems, Minneapolis, MN); and α-SMA (Sigma-Aldrich, St. Louis, MO). Mounted sections were viewed with an Axioskop Mot Plus microscope by using Axiovision software (Zeiss, Westlar, Germany). Vessel measurements were quantified using the same software.

### Vascular smooth muscle cell culture

Aortae were dissected, the adventitia stripped, and the vessel opened longitudinally. After collagenase digestion (1 mg/ml) for 10 minutes at 37°C applied to the endothelial surface, the remaining smooth muscle cells were grown by explant culture in standard conditions [17]. Immunostaining revealed >99% α-SMA positivity at day 14. Experiments were performed at passages 3 to 4. Cells were incubated for 24 hours in serum-free medium before agonist stimulation with TGF-β1 (4 ng/ml) or endothelin-1 (ET-1, 100 nmol/L). Cells were harvested using Buffer RLT (Qiagen, Crawley, UK) containing 10 μl/ml of 14.3 mmol/L β-ME or Laemmli blue buffer and stored at -70°C, or seeded into chamber slides for immunostaining.

### Immunostaining of vascular smooth muscle cells

Seeded cells were fixed with methanol at -20°C for 15 seconds or paraformaldehyde for 15 minutes and rinsed in PBS. After serum blocking, the cells were stained for 1 hour at room temperature with α-SMA, anti-smoothelin, anti-SM22α, or anti-β-galactosidase (Abcam), washed in PBS, and then incubated with the appropriate secondary antibody (Vector Laboratories) in PBS for 30 minutes. The slides were washed, mounted with Vectashield mounting medium containing DAPI (Vector Laboratories), and examined with an Axioskop Z fluorescence microscope (Zeiss).

### Reporter gene assay

The chemiluminescent β-galactosidase assay Galactolight Plus (Applied Biosystems, Foster City, CA) was used according to the manufacturer's instructions. In brief, equivalent numbers of vSMCs and fibroblasts in 96-well plates were lysed using the proprietary lysis buffer and incubated for 10 minutes. Then 10 μl was mixed with 70 μl reaction buffer and incubated for 1 hour; 100 μl of accelerator II was added automatically, and the luminescence was measured after 2 seconds using the Mithras LB 940 luminometer (Berthold, Wildbad, Germany). Assays were performed in triplicate.

### Assay of fibrillar collagen content

Newly synthesized acid-soluble collagens (types I to IV) from the heart or aorta were quantified by using the Sircol colorimetric assay (Biodye Science, Newtownabbey, UK) according to the manufacturer's instructions and analyzed using the Mithras LB 940 plate reader. Collagen concentrations were expressed as milligrams per milliliter. Data are expressed as mean ± SEM. Statistical comparisons were made by using Student's *t *test.

### Isometric tension measurement in isolated aortic rings

Mice thoracic descending aortae were washed in fresh Krebs buffer (119 mmol/L NaCl, 4.7 mmol/L KCl, 1.2 mmol/L MgSO4, 1.2 mmol/L KH2PO4, 11 mmol/L glucose, 25 mmol/L NaHCO3, 2.5 mmol/L CaCl2), and the loose connective tissue removed. Aortae were cut into paired 2- to 3-mm wide rings, which were mounted on two hooks in a 7 ml organ bath containing Krebs buffer at 37°C, continuously oxygenated with 95% O_2_/5% CO_2_. Isometric tension was measured with force-displacement transducers (Grass Instruments, Quincy, MA), and digitized using a multichannel recording system (Linton Instrumentation, Diss, Norfolk, UK) with MP100 acquisition unit and AcqKnowledge software (Biopac Systems, Goleta, CA). A resting tension of 500-550 mg was applied to the rings, which were then allowed to equilibrate for 60 minutes. In this period, tissues were washed out with Krebs buffer, and the applied tension readjusted at 15-minute intervals.

After the equilibration period, rings were contracted with cumulative doses of potassium chloride (KCl; 30 mmol/L and 80 mmol/L) until a stable contraction plateau was reached. Contractile responses were measured by recording changes in tension (milligrams). After washout, the tissues were allowed to reequilibrate for 30 minutes, and contractile dose-response curves were constructed using cumulative doses of phenylephrine (PE; 10^-9 ^to 10^-4 ^mol/L) and a stable analogue of thromboxane A_2 _(U46619; 10^-10 ^to 10^-4 ^mol/L) or ET-1 (10^-11 ^to 10^-7 ^mmol/L) with washout and equilibration after each dose response curve. In the relevant experiments, tissues were pretreated for 20 minutes with 2 mmol/L bosentan (a dual endothelin receptor antagonist) before contractile responses to ET-1 were measured. Data are expressed as mean ± SEM. A value of *P *< 0.05 was considered significant.

### Quantitative RT-PCR

Total RNA was extracted by using the RNeasy minikit (Qiagen) according to the manufacturer's instructions and quantified using the Nanodrop ND-8000 spectrophotometer (Thermo-Scientific, Wilmington, DE). The minimum 260:280 ratio was 1.90. RNA integrity numbers ranged from 8.8 to 10, measured on an Agilent 2100 Bioanalyzer (Agilent Technologies UK Limited, Stockport, UK); 600 ng of RNA was reverse transcribed using the Quantitect reverse transcription kit (Qiagen) and diluted fivefold with tRNA, 0.2 μg/ml. The real-time quantitative RT-PCR used 2 μl RNA in a 10 μl reaction volume by using Sensimix NoRef in a SYBR green-based assay (Quantace, London, UK) on a Rotorgene-6000 (Corbett Life Sciences, Sydney, Australia) under the following conditions: 95°C for 10 minutes, followed by 40 cycles of 95°C for 15 seconds, 57°C for 10 seconds, and 72°C for 5 seconds. Specific products and absence of primer dimers were confirmed by melt curve analysis. Copy numbers and assay efficiencies were derived from known copy number standard curves. Four stable reference genes: succinate dehydrogenase complex, subunit A (Sdha); ribosomal protein L13 (Rpl13); β-actin (ActB); and ubiquitin C (Ubc) were identified by using geNorm, and copy numbers were corrected using the computed normalization factor [18]. Primer sequences, written 5'-3', are referenced where appropriate, assay efficiency and R^2 ^follow: Sdha fwd TGTTCAGTTCCACCCCACA, rev TCTCCACGACACCCTTCTGT, 0.83, 0.991; Rpl13 fwd CAGTGAGATACCACACCAAGGTC, rev GTGCGAGCCACTTTCTTGT, 1.04, 0.998; ActB fwd CTAAGGCCAACCGTGAAAAG, rev ACCAGAGGCATACAGGGACA, 0.83, 0.999; Ubc fwd GAGTTCCGTCTGCTGTGTGA, rev TCACAAAGATCTGCATCGTCA, 0.93, 0.999; Pai-1 [19], 0.89, 1.000; mCol1a1 [19], 0.80, 0.993; Ctgf [19] 0.91, 0.998; Smtn (smoothelin) fwd CGAGGAGGCTGCAACTTTA, rev CTGCGCCATTAGCTGCTT, 0.96, 0.999, ETRA [20] 0.95, 0.999, ETRB [20], 0.94, 0.999, ET-1 fwd ACTTCTGCCACCTGGACATC, rev AGTTCTTTTCCTGCTTGGCA, 0.9, 0.999; Tagln fwd GATGGAACAGGTGGCTCAAT, rev TTCCATCGTTTTTGGTCACA, 0.94, 0.999.

### Floating collagen gel cultures

Experiments were performed as described previously [21]. In brief, 24-well tissue culture plates were precoated with 2.5% bovine serum albumin (BSA). Trypsinized smooth muscle cells were suspended in Molecular, Cellular, and Developmental Biology (MCDB) 131 medium (Invitrogen, Paisley, UK) and mixed with collagen solution (one part of 0.2 mol/L *N*-2-hydroxyethylpiperazine-*N'*-2-ethanesulfonic acid [HEPES], pH 8.0; four parts collagen [Vitrogen-100, 3 mg/ml, Cohesion Technologies, Palo Alto, CA], and five parts of 2× MCDB) yielding a final concentration of 80,000 cells/ml and 1.2 mg/ml collagen. Collagen/cell suspension (1 ml) was added to each well. After polymerization, gels were detached from wells by adding 1 ml of medium with or without TGF-β1 (4 ng/ml). Contraction of the gel was quantified by loss of gel weight and decrease in gel diameter over a 24-hour period. Comparison of collagen gel contraction was performed by using Student's *t *test. A value of *P *< 0.05 was considered statistically significant.

## Results

### Vascular fibrosis in transgenic mice is associated with increased TGF-β expression and signaling

**Figure 1:**
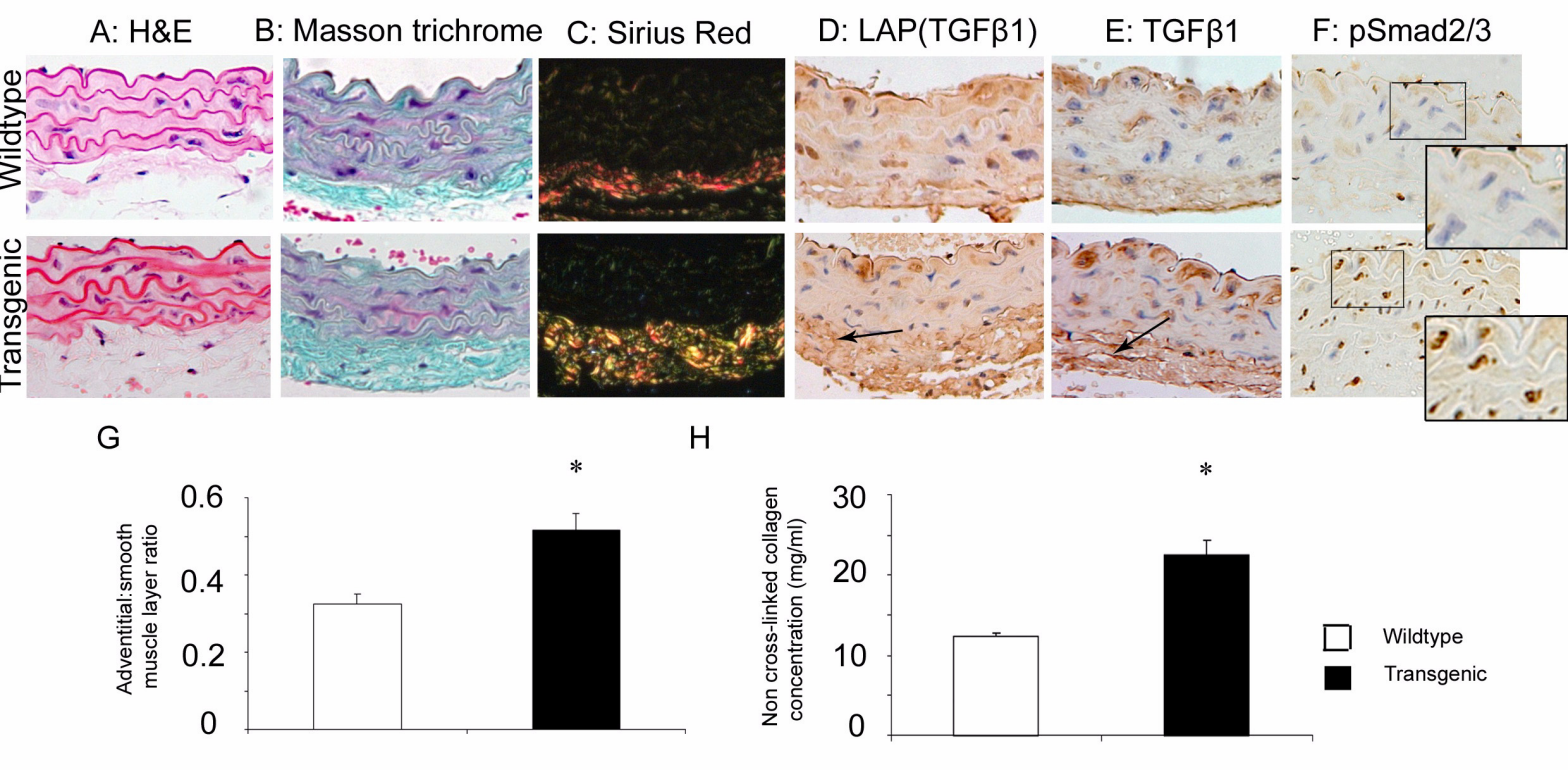
**Vascular fibrosis in transgenic mice is associated with increased TGF-β expression and signaling**. H&E, Masson trichrome, and picrosirius red (viewed under polarized light) staining of wild-type and transgenic thoracic aorta sections **(a-c)**. Smooth muscle layer architecture, elastic fibers, and smooth muscle layer are normal in the transgenic animals. However, adventitial collagen content is increased, and fibers are thicker in the transgenic animals on Masson trichrome and sirius red staining. LAP(TGF-β1) and free TGF-β1 expression is increased particularly in the adventitia (arrows) but also in smooth muscle cells **(d-f)**. Immunostaining for pSmad2/3 confirms increased nuclear translocation in the transgenic compared with the wild-type. Original magnification, ×20; representative images for panels **(a-f) **from transgenic (n = 6) and wild-type (n = 6) littermate sex-matched controls. **(g) **Serial measurements of adventitial and smooth muscle thickness show transgenic adventitial thickening and smooth muscle layer attenuation; summary data are expressed as mean ± SEM. *P < 0.05, from transgenic (n = 6) and wild-type (n = 6) littermate sex-matched controls. **(h) **Summary data from measurement of non-crosslinked collagen concentration compared with collagen standards (Sircol assay) show significantly higher collagen in transgenic animals compared with wild-type. Data are expressed as mean ± SEM.*P < 0.05, from transgenic (n = 8) and wild-type (n = 8) littermate sex-matched controls.

Figure 1a shows representative H&E-stained histologic sections of thoracic aortae from transgenic animals and wild-type littermate controls. The architecture of the medial smooth muscle layer was unchanged in the transgenic aortae, but adventitial thickness was increased. This difference is more apparent when stained with Masson trichrome, shown in Figure 1b, where the increased collagen content of the transgenic adventitia is demonstrated. Picrosirius red stain viewed with crossed polarized light shows the thicker yellow collagen fibers seen in the transgenic aortic tissue compared with the smaller orange-red fibers seen in the wild-type tissue (Figure 1c). Serial measurements of adventitial thickness on representative wild-type sections showed a mean ± SD of 19.3 ± 4.4 μm, and on transgenic sections, 27.37 ± 7.88 μm; *P *< 0.05. This is associated with attenuation of the smooth muscle layer, so that the adventitial/smooth muscle layer ratio is also increased in the transgenic animals (Figure 1g). Elastic van Giesson (EVG) staining revealed no differences in elastin distribution (data not shown). The histologic finding of increased adventitial collagen was confirmed by colorimetric Sircol assay for non-cross-linked collagen deposition in dissected thoracic aortae (mean transgenic, 22.5 ± 1.87 mg/ml; mean wild-type, 12.4 ± 0.45 mg/ml, *P *< 0.05), shown in Figure 1h. Consistent with previous studies that have shown increased TGF-β1 expression and activity in tissues from this transgenic mouse stain, immunostaining for latency-associated peptide for TGF-β1 (LAP(TGFβ1)) and TGF-β1 was increased in the aortic adventitia of transgenic animals, as expected. Increased nuclear translocation of pSmad 2/3 also occurred in transgenic mice in the smooth muscle layers, with a mean of 59.24 ± 6.43% positive nuclei in the transgenic animals compared with a mean of 39.42 ± 7.74% positive nuclei in the wild-type littermate controls (measured from 2 high-power fields for each mouse, n = 6 in each group; *P *< 0.001), confirming activation of Smad-dependent TGF-β signaling pathways in these cell lineages. Representative images are shown in Figure 1d-f. Overall, these results confirm that the increased levels of TGF-β in the extracellular matrix around large vessels in this strain activate signaling through TGF-β-dependent pathways in mesenchymal cell types, including vascular smooth muscle cells (vSMCs), and that this results in increased extracellular matrix deposition in vessel walls.

### Altered aortic ring vasoreactivity in transgenic mice

**Figure 2:**
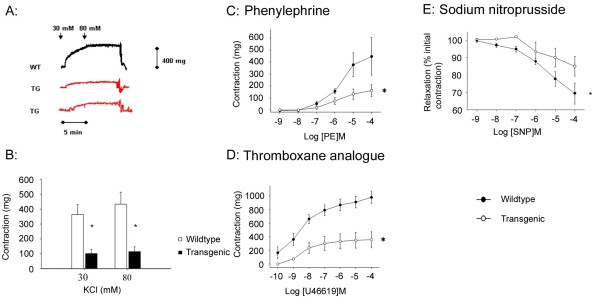
**Altered aortic ring vasoreactivity in transgenic mice**. **(a) **Representative vessel wall tension data from one wild-type (black trace) and two transgenic (red traces) aortic rings, showing attenuated contraction to two concentrations of KCl (30 and 80 mmol/L). **(b) **Summary data for wild-type (n = 7) and transgenic (n = 9) mice. Transgenic mouse aortae show significantly reduced contractile ability in response to direct stimulation of the smooth muscle cells with KCl at both low and high concentrations. **(c) **Phenylephrine (PE)-induced contraction is attenuated in transgenic aortic rings, and **(d) **U46619-induced (a stable thromboxane analogue) contraction is also reduced in the transgenic animal, plotted in response to cumulative concentrations of ligand. Vessels were obtained from WT (n = 8) and TG (n = 5) mice. **(e) **Vascular responses of aortic arteries isolated from WT (n = 9) and TG (n = 5) mice to cumulative concentrations of sodium nitroprusside (SNP) after preconstriction with U46619, data throughout figure are expressed as mean ± SEM; *P < 0.05.

To investigate whether the vessel wall fibrosis demonstrated in Figure 1 was associated with altered large vessel vasoreactivity in the Tβ RIIΔk-fib strain, we examined aortic ring responses to vasoactive agonists in isolated organ bath experiments. To elucidate key pathways that might be involved in regulating smooth muscle cell contraction, we used potassium chloride (KCl), which directly causes smooth muscle cell contraction, and a series of specific smooth muscle cell receptor agonists. Contractile responses to KCl were reduced in transgenic animals (*P *< 0.05; Figure 2a and 2b), and these were also reduced in response to vSMC stimulation with phenylephrine, an α-adrenoreceptor agonist (*P *< 0.05; Figure 2c), and U46619, a stable thromboxane analogue that acts through the thromboxane A_2 _receptor and is a potent vasoconstrictor in mice (*P *< 0.05; Figure 2d). The relaxation response with the NO donor sodium nitroprusside after precontraction with U46619 was also reduced in transgenic mice compared with wild-type (*P *< 0.05; Figure 2e). This finding may be confounded by the reduced contraction achieved by U46619, seen in Figure 2d, but is consistent with the hypothesis that this strain exhibits generalized arterial stiffness, and the reduction in dynamic response provides a clear functional correlate of this. The histologic changes demonstrated in Figure 1 can be correlated with the functional changes seen here, and with the hypothesis of TGF-β- mediated arterial stiffness.

### Cultured vascular smooth muscle cells from transgenic mice have a TGF-β- activated phenotype

**Figure 3:**
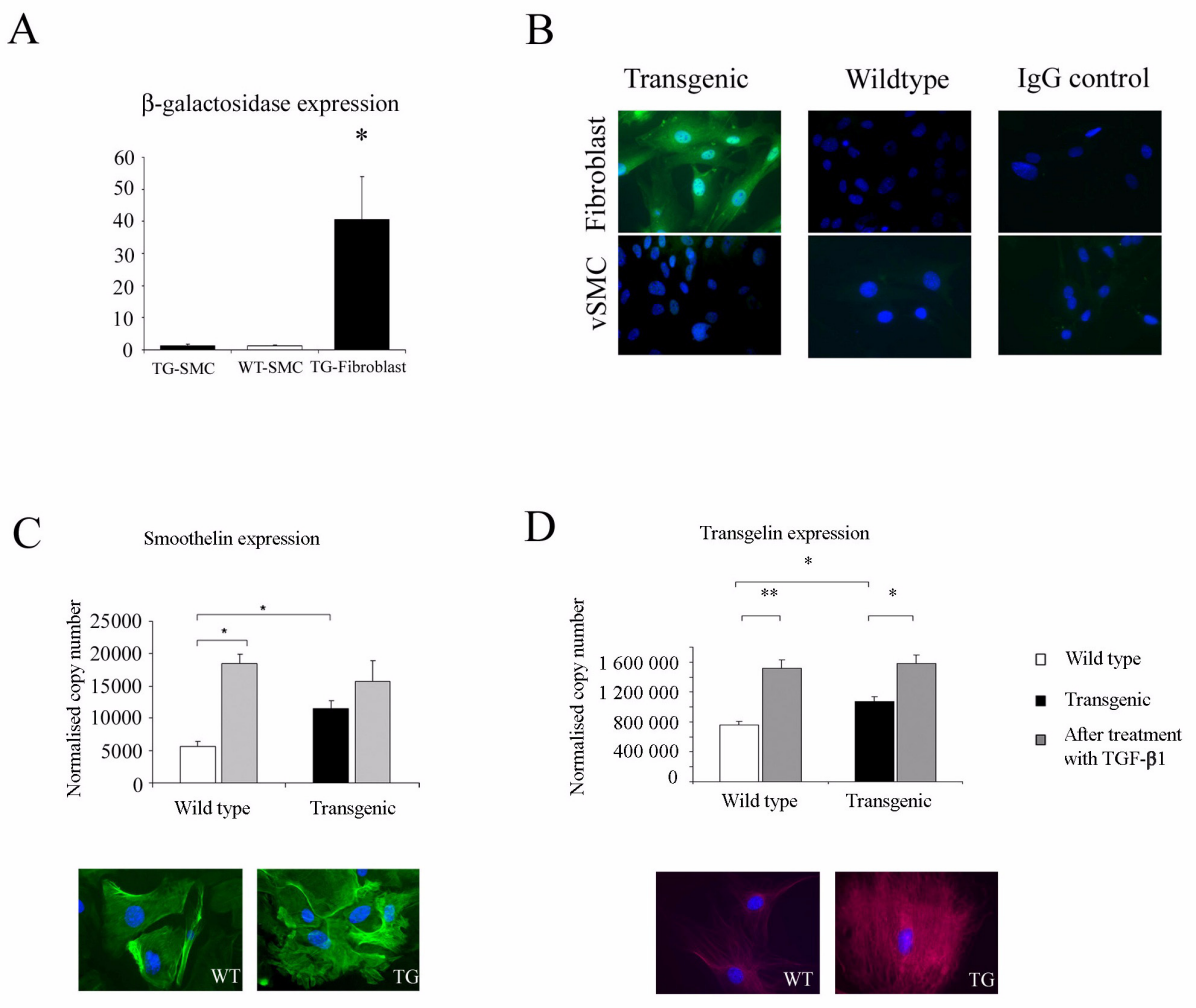
**Cultured vascular smooth muscle cells from transgenic mice have a TGF-β- activated phenotype**. **(a) **Reporter gene assay for β-galactosidase shows equal chemiluminescence in vSMCs from wild-type and transgenic mice. Transgenic fibroblasts from the same animals were used as a positive control. Data are expressed as mean ± SEM; *P < 0.05 from WT (n = 3) and TG (n = 3) animals. **(b) **β-Galactosidase immunostaining results comparing vSMCs and fibroblasts from wild-type and transgenic animals show negative staining in all vSMCs and positive staining only in transgenic fibroblasts. Images shown are representative of three independent experiments. **(c) **vSMCs from transgenic mice show increased expression of smoothelin gene by qPCR. Recombinant TGF-β1 induced smoothelin mRNA in wild-type vSMCs, but the response in cells from transgenic mice was attenuated. Immunostaining confirms constitutive overexpression of smoothelin in vSMCs from transgenic mice. **(d) **Data for transgelin, a second gene important for vSMC cytoskeletal function, show the same trends. Data are expressed as mean ± SEM; *P < 0.05, **P < 0.001; and are representative of three independent experiments examining four littermates for each condition. Original magnification, ×40.

In addition to structural changes with increased fibrous connective tissue in the aortic wall, we reasoned that our findings may reflect TGF-β- driven changes in vSMC properties reflecting an altered microenvironment *in vivo*. To explore this, early passage cultured aortic smooth muscle cells were analyzed before and after stimulation with TGF-β1 and ET-1, which has been shown to induce an overlapping cohort of profibrotic genes in other cell types [22]. No significant difference was found in growth curves over 48 hours, α-SMA protein expression or distribution between wild-type vSMCs, or those from transgenic animals (data not shown). A quantitative reporter gene assay for β-galactosidase activity confirmed that wild-type vSMCs and those from transgenic animals had equal chemiluminescence and hence that the transgene was not expressed in these cells. These results were confirmed on immunofluorescent staining of vSMCs from wild-type and transgenic animals, by using transgenic fibroblasts as a positive control (Figure 3a, b). Smoothelin gene and protein expression was elevated in cells from transgenic animals (Figure 3c). This molecular hallmark of contractile vSMCs was previously reported to be regulated by TGF-β [23]. Although exogenous administration of TGF-β1 to wild-type cells resulted in upregulation of smoothelin gene expression, the cells from transgenic animals did not significantly induce further gene expression, despite elevated basal expression at comparable levels to TGF-β1-activated wild-type cells. A similar, but more pronounced pattern was demonstrated for transgelin gene expression, another important cytoskeletal component in vSMCs, with significantly enhanced baseline expression in vSMCs from transgenic mice (Figure 3d). Together, these observations suggest a constitutive activation of TGF-β- regulated gene expression in vSMCs of transgenic mice that is analogous to previously reported abnormalities in expression of TGF-β- regulated genes in dermal fibroblasts of this mouse strain [12]. These findings are consistent with the immunostaining data for pSmad2/3 shown in Figure 1f. It is noteworthy that some other TGF-β- regulated genes less specific to vSMCs did not show this pattern of overexpression. Thus, Pai-1, Ctgf, and Col1a1 were not significantly different at RNA level in cells from transgenic animals when compared with the wild-type and were equivalently induced by recombinant TGF-β1. For example, Pai-1 was strongly induced with recombinant TGF-β1, mean fold change 5.3 times baseline (*P *< 0.05) in cells from both wild-type and transgenic animals. Induction by ET-1 was comparable at 5.6- and 6.8-fold, respectively (*P *< 0.05). These findings contrast with the results from skin fibroblasts from this mouse strain, in which these genes were significantly upregulated, and suggest that whereas some of the molecular phenotype is shared between fibroblasts and vSMCs in this transgenic strain, important lineage-specific differences may exist. This is not surprising, considering that transgene expression is regulated by a fibroblast-specific promoter that would be expected to lead to direct perturbation of TGF-β signaling and responses in fibroblasts but not in other cell types.

### Vascular smooth muscle cells from Tβ RIIΔk-fib transgenic mice show enhanced remodeling of floating type I collagen gel lattices

**Figure 4:**
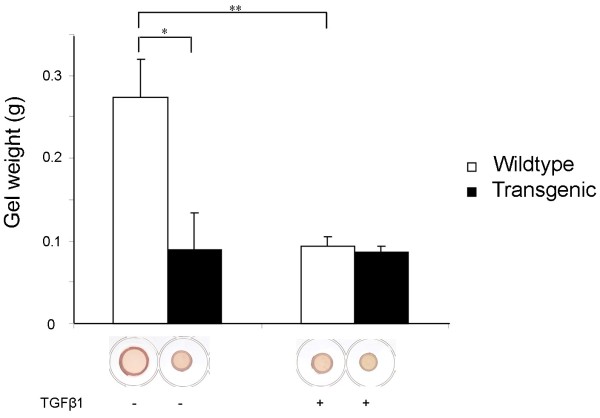
**Vascular smooth muscle cells from Tβ RIIΔk-fib mice show enhanced remodeling of floating type I collagen gel lattices**. vSMCs from transgenic animals promoted more contraction of free-floating collagen lattices, resulting in gels of reduced diameter and weight when compared with wild-type. Induction by exogenous TGF-β1 resulted in further contraction by wild-type cells, but cells from transgenic mice were refractory to further induction. Data are expressed as mean ± SEM;*P < 0.05, **P < 0.001, and are representative of three independent experiments examining three littermates for each condition.

Pooled data from a series of independent contraction assays using type I collagen gel lattices delineated an important functional effect of this activated phenotype. Figure 4 shows contraction assays from vSMCs of transgenic mice compared with wild-type littermates. vSMCs from transgenic mice promoted more contraction of free-floating lattices, resulting in gels of reduced diameter and weight, consistent with an activated profibrotic phenotype. Exogenous TGF-β1 induced further contraction by wild-type cells, but cells from transgenic animals were refractory to further induction (Figure 4).

### Perturbed endothelin receptor expression and function in transgenic vascular smooth muscle cells

**Figure 5:**
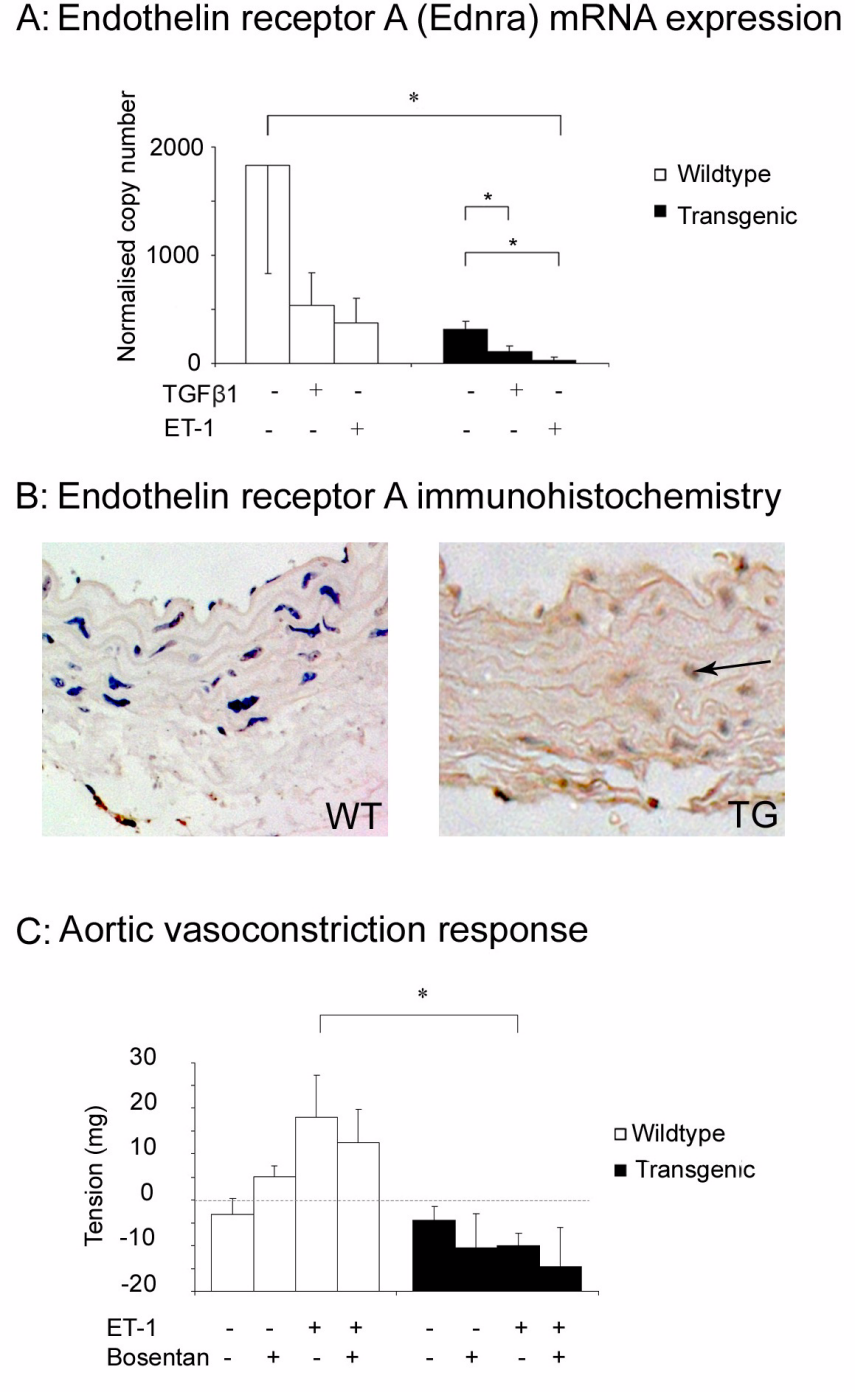
**Perturbed endothelin receptor expression and function in vascular smooth muscle cells from transgenic animals**. **(a, b) **vSMCs from transgenic mice have reduced expression of ETRA mRNA and protein when compared with wild-type cells. Exogenous administration of TGF-β or ET-1 to cells from both wild-type and transgenic animals further suppressed ETRA expression. Data are representative of three independent experiments examining four littermates for each condition and are expressed as mean ± SEM,*P < 0.05, **P < 0.001. **(c) **Vasoconstrictor response of aortic rings to ET-1 was attenuated in transgenic mice. Bosentan attenuates the response in both wild-type and transgenic mice. Representative data from dose response curves of WT (n = 6) and TG (n = 6) aortic rings before (-) and after (+) response to 10^-8 ^mol/L ET-1 concentrations, before (-) and after (+) bosentan 2 μmol/L pretreatment. Data are expressed as mean ± SEM; *P < 0.05.

Previous work suggested important functional cross-talk between TGF-β and ET-1 that may be relevant to fibrosis and potentially important in the pathogenesis of SSc and its vascular complications. We therefore explored endothelin-1 (ET-1) and endothelin receptor A (ETRA) and B (ETRB) mRNA expression in vSMCs with quantitative PCR. As expected from previous reports, expression of ET-1 and ETRA was noted in wild-type vSMCs, but very low expression of ETRB (data not shown) was found [24]. vSMCs from transgenic mice have reduced expression of ETRA mRNA and protein when compared with wild-type cells, shown in Figure 5a and 5b. It previously was reported that treatment of vSMCs with either TGF-β1 or ET-1 downregulates ETRA expression. Our results were consistent with this: exogenous administration of TGF-β or ET-1 to cells from both wild-type and transgenic mice further suppressed ETRA mRNA expression (Figure 5a). The finding of reduced expression of ETRA in vSMCs is consistent with *in vivo *upregulation of their ligands and suggests that fibroblast-derived mediators may be critical for the development of this altered vSMC phenotype. No significant differences in ET-1 expression were seen between vSMC cultures from wild-type or transgenic mice, consistent with the predominantly endothelial expression of ET-1.

To investigate the functional consequences of altered endothelin receptor expression in this transgenic strain, we measured isometric tension in aortic rings from wild-type and transgenic animals. Contractile responses to ET-1 were lower in the transgenic aortae when compared with the wild-type (Figure 5c). Moreover, a consistent trend was noted to vasodilation in the transgenic aortae, which may reflect the altered endothelin receptor A/B balance in these samples. Pretreatment with a potent endothelin receptor inhibitor (bosentan) reduced the responsiveness of wild-type aortic rings to ET-1 but, as expected, had little effect on responses in the transgenic aortae.

### Myocardial fibrosis in TβRIIΔk-fib transgenic mice

**Figure 6:**
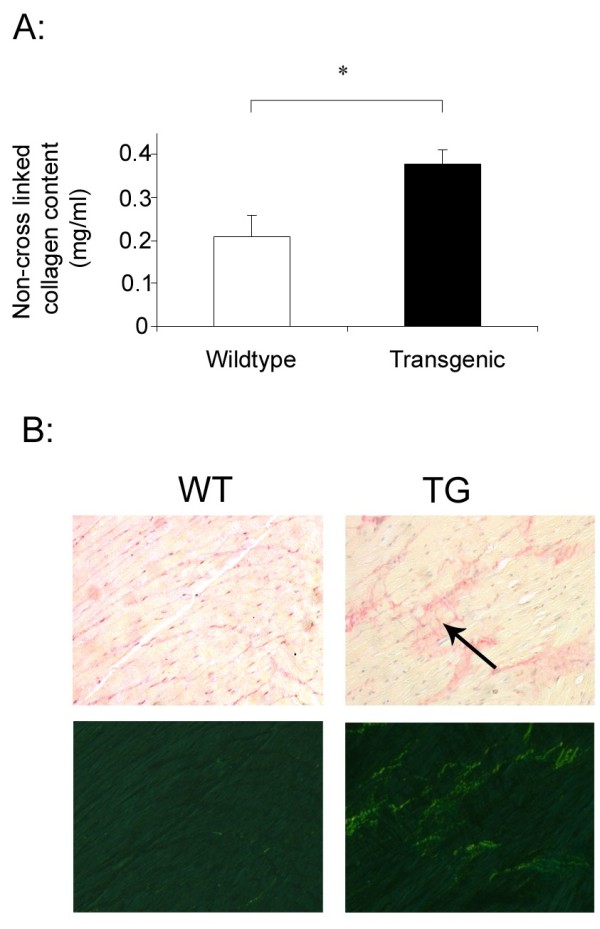
**Myocardial fibrosis in Tβ RIIΔk-fib transgenic mice**. **(a) **Transgenic animals have significantly higher non-crosslinked collagen content when compared with sex-matched littermate controls (n = 10). Data are expressed as mean ± SEM, *P < 0.05. **(b) **Picrosirius red stains of the left ventricles of WT and TG animals viewed under bright-field and polarized light microscopy. Transgenic animals have a diffuse increase in collagen deposition within the myocardial interstitium. Original magnification, ×20, representative images from transgenic (n = 6) and wild-type (n = 6) littermate sex-matched controls.

Another important manifestation of SSc is interstitial myocardial fibrosis. In this transgenic strain, we predicted that myocardial fibrosis would occur and may reflect an altered in vivo hemodynamic phenotype in this mouse strain as well as potentially intrinsic fibrosis within the heart. Indeed, transgenic animals showed evidence of myocardial fibrosis on quantitative measurement of non-cross-linked collagen content and on picrosirius red staining. These findings are summarized in Figure 6: picrosirius red stain is viewed with both bright-field and polarized-light microscopy. No inflammatory cell infiltrate was evident on H&E staining, and findings were similar for the left and right ventricles. These findings provide evidence that altered aortic dynamics and altered fibroblast interactions with smooth muscle or cardiac muscle cells result in cardiac fibrosis.

## Discussion

In this study, we examined the systemic vasculature in a mouse model of SSc in which the primary defect is fibroblast-specific perturbation of TGF-β signaling. We defined, for the first time in this strain, a structural vasculopathy with adventitial fibrosis and smooth muscle attenuation in the thoracic aorta and further demonstrated altered vasoreactivity in isolated vessel preparations *in vitro*. Smooth muscle cell cultures show upregulation of TGF-β- dependent genes, and cardiac fibrosis is evident. Our work complements earlier studies of skin and lung fibrosis in this transgenic mouse strain.

Previous studies of cultured cells derived from this transgenic mouse strain have focused on the properties of fibroblasts [12]. Exploration of the biochemical and functional properties of vSMCs provides important insight into the potential pathogenic mechanisms of vascular fibrosis. The lineage-specific nature of transgene expression precludes an intrinsic perturbation of TGF-β signaling in vSMCs, as they do not express the nonsignaling type II TGF-β receptor, confirmed in Figure 3a and 3b. This explains the greater responsiveness for cardinal TGF-β-regulated transcripts that we observe in vSMCs compared with dermal fibroblasts [12]. This is consistent with balanced upregulation of TGF-β signaling in fibroblasts *in vitro*, whereas the activated phenotype of explanted vSMCs reflects previous *in vivo *activation by extracellular TGF-β. Thus, alterations in vascular smooth muscle cell function are likely to reflect paracrine effects mediated by transgenic fibroblasts. This is concordant with the altered epithelial cell phenotype observed in the lungs of this mouse strain in our studies of lung fibrosis [19], which also is attributed to bystander effects of fibroblast-dependent increased local levels of active TGF-β ligand. The alterations in endothelin signaling within the vSMCs of the Tβ RIIΔk-fib strain are reminiscent of those seen in SSc fibroblasts, which have low ETRA expression in the context of high ET-1 levels. Previous work confirmed the importance of functional cross-talk between TGF-β and ET-1 [25,26] in SSc pathogenesis.

Our findings extend and validate data from other TGF-β- dependent animal models of SSc. For example a rapidly progressive vasculopathy is described in the caveolin-1 knockout mouse, which occurs in part because of uncontrolled endothelial proliferation, alterations in vasomotor tone, and a fibrotic phenotype associated with increased signaling through the TGF-β axis [27,28], and second, the Tβ RI^CA ^Cre-ER mouse strain in which constitutive activation of the Tβ RI in fibroblasts results in fibrotic thickening of small vessels in the lung and kidney but histologically normal large vessels and heart [29]. The heterozygous TSK-1 mouse, which carries a 30- to 40-kb genomic duplication in the *fibrillin-1 *gene, has marked hyperplasia of loose connective tissue around the thoracic aorta [30] and altered aortic hemodynamics *ex vivo *suggestive of endothelial dysfunction [31]. These models allow important investigation into the link between endothelial cell dysfunction and fibrosis but do not address the more chronic background vasculopathy that is a hallmark of SSc and may underlie susceptibility to important clinical complications, including PAH and SRC.

In this study, structural and dynamic alterations in large vessels are evident. Abnormalities in elasticity and compliance are most evident in patients with diffuse cutaneous SSc [2,6]. These result in a phenotype of arterial stiffness, which is usually considered to have independent predictive value for cardiovascular events. Whether SSc predisposes to increased atherosclerotic risk remains in question: some reports exist of increased propensity to peripheral vascular disease in limited cutaneous SSc, but an association of coronary artery disease with SSc has not been consistently demonstrated [7,32,33]. Examination of the microvascular structure in this model in the future, particularly within the vascular beds of the lung, kidney, and dermis, is likely to provide further insight into the molecular basis of vasculopathy in fibrotic disorders such as SSc.

Potential mechanistic parallels exist between the Tβ RIIΔk-fib mouse strain and human Loeys-Dietz syndrome, in which mutations in Tβ RI and Tβ RII result in paradoxical increased expression of TGF-β- regulated proteins and signaling pathways. The fibroblast-specific nature of transgene expression is a likely explanation for the absence of greater phenotypic similarity in this mouse strain. In animal models of essential hypertension, arterial stiffness does not develop because of structural modifications of the vessel walls with redistribution of the mechanical load toward elastic materials. Alterations in the capacities of these remodeling processes may explain the spectrum of arterial disease seen in Marfan syndrome, Loeys-Dietz syndrome, SSc, and hypertension; fibrillin and TGF-β metabolism are implicated in all [11,34,35].

The significance of myocardial fibrosis in the Tβ RIIΔk-fib strain is unclear. It may result from altered pulmonary and systemic hemodynamics or as a primary process from excessive TGF-β due to the genetic defect in the fibroblasts present within the myocardium. It is possible that an initial response to altered vascular dynamics results in increased fibroblast activity in the myocardium and hence higher expression of the transgene and upregulation of TGF-β. Autopsy studies have revealed evidence of myocardial interfascicular fibrosis and contraction-band necrosis in patients with SSc, and myocardial involvement is an adverse prognostic feature of this condition [36]. The presence of increased cardiac collagen in this mouse strain strengthens its place as a useful disease model.

Limitations of this study include the challenge of directly extrapolating biochemical and functional results from a mouse model to a complex multisystem disease such as SSc. Moreover, it is challenging to separate primary effects of an alteration of fibroblast-derived TGF-β from those that are due to altered vascular smooth muscle cell properties. Differences may exist between *in vivo *mechanisms and the properties of explanted cells in tissue culture or isolated organ bath preparations, although this method was selected as it provides one of the most physiologic platforms for studies of vasoreactivity *ex vivo*. As expected from the published literature [37], murine aortic rings were only weakly responsive to endothelin, in contrast to vessels from other species. Where the tension axis falls below zero in Figure 5c, suggesting vasodilation, we speculate that this relates to unopposed vasodilator effect of type B endothelin receptors. However, although consistent, this effect did not reach statistical significance. Technical limitations of this study include the need to perform studies on relatively small numbers of mice and the measurement of mRNA expression levels that may not correlate with function or activity of encoded protein.

## Conclusions

In conclusion, our study delineates a TGF-β- dependent macrovascular phenotype in this transgenic mouse strain associated with vessel wall fibrosis and altered vSMC properties, including altered TGF-β and ET-1 responses. These results provide support for a potential link between perturbed TGF-β and ET-1 bioactivity and cardiovascular manifestations of human SSc.

## Abbreviations

α- SMA: α-smooth muscle actin; β-ME: β- mercaptoethanol; BSA: bovine serum albumin; Col1a1: collagen: type 1: alpha-1 mRNA; Ctgf: connective tissue growth factor; DNA: deoxyribonucleic acid; ETRA: endothelin receptor A; ETRB: endothelin receptor B; ET-1: endothelin-1; EVG: elastin van Giesson; H&E: hematoxylin and eosin; Kb: kilobase; KCl: potassium chloride; LAP(TGFβ1): latency-associated peptide to TGF-β1; mRNA: messenger ribonucleic acid; NO: nitric oxide; PAH: pulmonary arterial hypertension; PBS: phosphate-buffered saline; PCR: polymerase chain reaction; PE: phenylephrine; pSmad2/3: phospho-Smad2/3; qPCR: quantitative polymerase chain reaction; RNA: ribonucleic acid; RT-PCR: reverse transcription polymerase chain reaction; SD: standard deviation; SEM: standard error of mean; Smtn: smoothelin mRNA; SNP: sodium nitroprusside; SRC: scleroderma renal crisis; SSc: systemic sclerosis: scleroderma; Tagln: transgelin mRNA; Tβ RI: transforming growth factor beta receptor type I; Tβ RII: transforming growth factor beta receptor type II; Tβ RIIΔk-fib: fibroblast-specific kinase-deficient TGF-β type II receptor strain; TG: transgenic; TGF-β: transforming growth factor beta; TSK-1: tight-skin-1 mouse; vSMC: vascular smooth muscle cell; WT: wild-type.

## Competing interests

The authors declare that they have no competing interests.

## Authors' contributions

CPD had full access to all of the data in the study and takes responsibility for the integrity of the data and the accuracy of the data analysis. ECD-S, DJA, and CPD participated in the study design. ECD-S, AD, KK, and XS-W participated in the acquisition of data. ECD-S, AD, Khan, XS-W, and CPD participated in the analysis and interpretation of data. ECD-S, DJA, and CPD prepared the manuscript. ECD-S was responsible for statistical analysis.

## References

[B1] Fleming JN Schwartz SM The pathology of scleroderma vascular disease Rheum Dis Clin North Am 2008 34 41 55 vi 10.1016/j.rdc.2008.01.001 18329531

[B2] Cheng KS Tiwari A Boutin A Denton CP Black CM Morris R Hamilton G Seifalian AM Carotid and femoral arterial wall mechanics in scleroderma Rheumatology (Oxford) 2003 42 1299 1305 10.1093/rheumatology/keg371 12777634

[B3] Cheng KS Tiwari A Boutin A Denton CP Black CM Morris R Seifalian AM Hamilton G Differentiation of primary and secondary Raynaud's disease by carotid arterial stiffness Eur J Vasc Endovasc Surg 2003 25 336 341 10.1053/ejvs.2002.1845 12651172

[B4] Sfikakis PP Papamichael C Stamatelopoulos KS Tousoulis D Fragiadaki KG Katsichti P Stefanadis C Mavrikakis M Improvement of vascular endothelial function using the oral endothelin receptor antagonist bosentan in patients with systemic sclerosis Arthritis Rheum 2007 56 1985 1993 10.1002/art.22634 17530638

[B5] Moyssakis I Gialafos E Vassiliou V Taktikou E Katsiari C Papadopoulos DP Sfikakis PP Aortic stiffness in systemic sclerosis is increased independently of the extent of skin involvement Rheumatology 2005 44 251 254 10.1093/rheumatology/keh478 15546962

[B6] Timár O Soltész P Szamosi S Dér H Szántó S Szekanecz Z Szücs G Increased arterial stiffness as the marker of vascular involvement in systemic sclerosis J Rheumatol 2008 35 1329 1333 18484693

[B7] Akram MR Handler CE Williams M Carulli M Andron M Black CM Denton CP Coghlan JG Angiographically proven coronary artery disease in scleroderma Rheumatology 2006 45 1395 1398 10.1093/rheumatology/kel120 16606654

[B8] Grainger D Kemp PR Metcalfe JC Liu AC Lawn RM Williams NR Grace AA Schofield PM Chauhan A The serum concentration of active transforming growth factor-beta is severely depressed in advanced atherosclerosis Nature Medicine 1995 1 74 79 10.1038/nm0195-74 7584958

[B9] Leask A Scar wars: is TGFβ the phantom menace in scleroderma? Arthritis Res Ther 2006 8 213 219 10.1186/ar1976 16774692 PMC1779423

[B10] Neptune ER Frischmeyer PA Arking DE Myers L Bunton TE Gayraud B Ramirez F Sakai LY Dietz HC Dysregulation of TGF-beta activation contributes to pathogenesis in Marfan syndrome Nat Genet 2003 33 407 411 10.1038/ng1116 12598898

[B11] Loeys BL Chen J Neptune ER Judge DP Podowski M Holm T Meyers J Leitch CC Katsanis N Sharifi N Lauren Xu F Myers LA Spevak PJ Cameron DE De Backer J Hellemans J Chen Y Davis EC Webb CL Kress W Coucke P Rifkin DB De Paepe AM Dietz HC A syndrome of altered cardiovascular, craniofacial, neurocognitive and skeletal development caused by mutations in TGFBR1 or TGFBR2 Nat Genet 2005 37 275 281 10.1038/ng1511 15731757

[B12] Denton CP Lindahl GE Khan K Shiwen X Ong VH Gaspar NJ Lazaridis K Edwards DR Leask A Eastwood M Leoni P Renzoni EA Bou Gharios G Abraham DJ Black CM Activation of key profibrotic mechanisms in transgenic fibroblasts expressing kinase-deficient type II TGFbeta receptor J Biol Chem 2005 280 16053 16065 10.1074/jbc.M413134200 15708853

[B13] Antoniv TT De Val S Wells D Denton CP Rabe C de Crombrugghe B Ramirez F Bou-Gharios G Characterization of an evolutionarily conserved far-upstream enhancer in the human alpha 2(I) collagen (COL1A2) gene J Biol Chem 2001 276 21754 21764 10.1074/jbc.M101397200 11279244

[B14] Denton CP Zheng B Shiwen X Zhang Z Bou-Gharios G Eberspaecher H Black CM de Crombrugghe B Activation of a fibroblast-specific enhancer of the proalpha2(I) collagen gene in tight-skin mice Arthritis Rheum 2001 44 712 722 10.1002/1529-0131(200103)44:3<712::AID-ANR121>3.0.CO;2-1 11263787

[B15] Denton CP Zheng B Evans LA Shi-wen X Ong VH Fisher I Lazaridis K Abraham DJ Black CM de Crombrugghe B Fibroblast-specific expression of a kinase-deficient type II transforming growth factor beta (TGFbeta) receptor leads to paradoxical activation of TGFbeta signaling pathways with fibrosis in transgenic mice J Biol Chem 2003 278 25109 25119 10.1074/jbc.M300636200 12707256

[B16] Denton CP Khan K Hoyles RK Shiwen X Leoni P Chen Y Eastwood M David JA Inducible lineage-specific deletion of Tβ RII in fibroblasts defines a pivotal regulatory role during adult skin wound healing J Invest Dermatol 2009 129 194 204 10.1038/jid.2008.171 18563179

[B17] Rong JX Shapiro M Trogan E Fisher EA Transdifferentiation of mouse aortic smooth muscle cells to a macrophage-like state after cholesterol loading Proc Natl Acad Sci USA 2003 100 13531 13536 10.1073/pnas.1735526100 14581613 PMC263848

[B18] Vandesompele J De Preter K Pattyn F Poppe B Van Roy N De Paepe A Speleman F Accurate normalization of real-time quantitative RT-PCR data by geometric averaging of multiple internal control genes Genome Biol 2002 3 RESEARCH0034 10.1186/gb-2002-3-7-research0034 PMC126239 12184808

[B19] Hoyles RK Khan K Shiwen X Howat SL Lindahl GE Leoni P du Bois RM Wells AU Black CM Abraham DJ Denton CP Fibroblast-specific perturbation of transforming growth factor beta signalling provides insight into potential pathogenic mechanisms of scleroderma-associated lung fibrosis: exaggerated response to alveolar epithelial injury in a novel mouse model Arthritis Rheum 2008 58 1175 1188 10.1002/art.23379 18383385

[B20] Machado FS Desruisseaux Nagajyothi MS Kennan RP Hetherington HP Wittner M Weiss LM Lee SC Scherer PE Tsuji M Tanowitz HB Endothelin in a murine model of cerebral malaria Exp Biol Med (Maywood) 2006 231 1176 1181 16741072

[B21] Shi-Wen X Chen Y Denton CP Eastwood M Renzoni EA Bou-Gharios G Pearson JD Dashwood M du Bois RM Black CM Leask A Abraham DJ Endothelin-1 promotes myofibroblast induction through the ETA receptor via a rac/phosphoinositide 3-kinase/Akt-dependent pathway and is essential for the enhanced contractile phenotype of fibrotic fibroblasts Mol Biol Cell 2004 15 2707 2719 10.1091/mbc.E03-12-0902 15047866 PMC420095

[B22] Shi-wen X Kennedy L Renzoni EA Bou-Gharios G du Bois RM Black CM Denton CP Abraham DJ Leask A Endothelin is a downstream mediator of profibrotic responses to transforming growth factor β in human lung fibroblasts Arthritis Rheum 2007 56 4189 4194 10.1002/art.23134 18050250

[B23] Loop FT van der Schaart G Timmer ED Ramaekers FC van Eys GJ Smoothelin, a novel cytoskeletal protein specific for smooth muscle cells J Cell Biol 1996 134 401 411 10.1083/jcb.134.2.401 8707825 PMC2120883

[B24] Davenport AP O'Reilly G Kuc RE Endothelin ETA and ETB mRNA and receptors expressed by smooth muscle in the human vasculature: majority of the ETA sub-type Br J Pharmacol 1995 114 1110 1116 7620699 10.1111/j.1476-5381.1995.tb13322.x PMC1510347

[B25] Abraham DJ Vancheeswaran R Dashwood MR Rajkumar VS Pantelides P Xu SW du Bois RM Black CM Increased levels of endothelin-1 and differential endothelin type A and B receptor expression in scleroderma-associated fibrotic lung disease Am J Pathol 1997 151 831 841 9284832 PMC1857854

[B26] Horstmeyer A Licht C Scherr G Eckes B Krieg T Signalling and regulation of collagen I synthesis by ET-1 and TGF-beta1 FEBS J 2005 272 6297 6309 10.1111/j.1742-4658.2005.05016.x 16336267

[B27] Razani B Engelman JA Wang XB Schubert W Zhang XL Marks CB Macaluso F Russell RG Li M Pestell RG Di Vizio D Hou H Jr Kneitz B Lagaud G Christ GJ Edelmann W Lisanti MP Caveolin-1 null mice are viable but show evidence of hyperproliferative and vascular abnormalities J Biol Chem 2001 276 38121 38138 10.1074/jbc.M008340200 11457855

[B28] Wunderlich C Schober K Schmeisser A Heerwagen C Tausche AK Steinbronn N Brandt A Kasper M Schwencke C Braun-Dullaeus RC Strasser RH The adverse cardiopulmonary phenotype of caveolin-1 deficient mice is mediated by a dysfunctional endothelium J Mol Cell Cardiol 2008 44 938 947 10.1016/j.yjmcc.2008.02.275 18417152

[B29] Sonnylal S Denton CP Zheng B Keene DR He R Adams HP Vanpelt CS Geng YJ Deng JM Behringer RR de Crombrugghe B Postnatal induction of transforming growth factor beta signaling in fibroblasts of mice recapitulates clinical, histologic, and biochemical features of scleroderma Arthritis Rheum 2007 56 334 344 10.1002/art.22328 17195237

[B30] Akita M Lee SH Kaneko K Electron microscopic observations of elastic fibres in the lung and aorta of tight-skin and beta-aminopropionitrile-fed mice Histol Histopathol 1992 7 39 45 1576433

[B31] Marie I Bény JL Endothelial dysfunction in murine model of systemic sclerosis: tight-skin mice 1 J Invest Dermatol 2002 119 1379 1387 10.1046/j.1523-1747.2002.19614.x 12485443

[B32] Youssef P Brama T Englert H Bertouch J Limited scleroderma is associated with increased prevalence of macrovascular disease J Rheumatol 1995 22 469 472 7783063

[B33] Ho ML Veale D Eastmond C Nuki G Belch J Macrovascular disease and systemic sclerosis Ann Rheum Dis 2000 59 39 43 10.1136/ard.59.1.39 10627425 PMC1752979

[B34] Chung AWY Yang HHC Yeung KA van Breemen C Mechanical and pharmacological approaches to investigate the pathogenesis of Marfan syndrome in the abdominal aorta J Vasc Res 2008 45 314 322 10.1159/000113603 18212506

[B35] Bézie Y Lacolley P Laurent S Gabella G Connection of smooth muscle cells to elastic lamellae in aorta of spontaneously hypertensive rats Hypertension 1998 32 166 169 9674655 10.1161/01.hyp.32.1.166

[B36] Coghlan JG Mukerjee D The heart and pulmonary vasculature in scleroderma: clinical features and pathobiology Curr Opin Rheumatol 2001 13 495 499 10.1097/00002281-200111000-00008 11698727

[B37] Widmer CC Mundy AL Kretz M Barton M Marked heterogeneity of endothelin-mediated contractility and contraction dynamics in mouse renal and femoral arteries Exp Biol Med (Maywood) 2006 231 777 781 16740998

